# Atypical anti-glomerular basement membrane disease with anti-GBM antibody negativity and ANCA positivity: a case report

**DOI:** 10.1186/s12882-021-02232-1

**Published:** 2021-02-05

**Authors:** Na Guo, Qinghua Yin, Song Lei, Yanjun He, Ping Fu

**Affiliations:** 1 Kidney Research Institute, Division of Nephrology, West China Hospital, Sichuan University, Chengdu, 610041 China; 2 Department of Pathology, West China Hospital, Sichuan University, Chengdu, 610041 China

**Keywords:** Anti-glomerular basement membrane disease, Goodpasture syndrome, Anti-neutrophil cytoplasmic antibody, Case report

## Abstract

**Background:**

Anti-glomerular basement membrane (anti-GBM) disease is an organ-specific autoimmune disease that involves the lung and kidneys and leads to rapid glomerulonephritis progression, with or without diffuse alveolar hemorrhage, and even respiratory failure. Classic cases of anti-GBM disease are diagnosed based on the presence of the anti-GBM antibody in serum samples and kidney or lung biopsy tissue samples. However, atypical cases of anti-GBM disease are also seen in clinical practice.

**Case presentation:**

We herein report the rare case of a patient with atypical anti-GBM disease whose serum was negative for the anti-GBM antibody but positive for the myeloperoxidase (MPO) anti-neutrophil cytoplasmic antibody (p-ANCA) and another atypical ANCA. Laboratory test results showed severe renal insufficiency with a creatinine level of 385 μmol/L. Renal biopsy specimen analysis revealed 100% glomeruli with crescents; immunofluorescence showed immunoglobulin G (IgG) linearly deposited alongside the GBM. Finally, the patient was discharged successfully after treatment with plasmapheresis, methylprednisolone and prednisone.

**Conclusion:**

This patient, whose serum was negative for the anti-GBM antibody but positive for p-ANCA and another atypical ANCA, had a rare case of anti-GBM disease. Insights from this unusual case might help physicians diagnose rare forms of glomerulonephritis and treat affected patients in a timely manner.

## Background

Anti-glomerular basement membrane (anti-GBM) disease, or Goodpasture syndrome, is an organ-specific autoimmune disease that involves the lung and kidneys and leads to the rapid progression of glomerulonephritis, with or without diffuse alveolar hemorrhage, and even respiratory failure. Classic cases of anti-GBM disease are diagnosed based on the presence of anti-GBM antibodies in serum samples and kidney or lung biopsy tissue samples [1]. However, atypical cases of anti-GBM disease are also seen in clinical practice [2, 3]. The case of the patient presented here, whose biopsy specimen analysis showed 100% crescents and immunoglobulin G (IgG) linearly deposited alongside the GBM and who repeatedly tested negative for serum anti-GBM antibodies but positive for the anti-myeloperoxidase (MPO) antibody (p-ANCA) and another atypical ANCA, is unusual. Insights from this unusual case might help clinicians identify unusual features of glomerulonephritis and administer proper treatments in a timely manner.

## Case presentation

A 48-year-old Chinese woman was administered a blood-activating and stasis-eliminating compound by a local physician for ankle pain. A “blood-activating and stasis-eliminating compound” is a type of traditional medicine that can be used as an analgesic and antipyretic. The ingredients of this blood-activating and stasis-eliminating compound include safflower, dog ridge (made), mistletoe, herba lycopi leaf, spatholobi, caulis trachelospermi, cocklebur, *Cyperus rotundus*, *Periploca sepium* Bge, and natural copper (calcined). It does not cause an autoimmune response, such as rash or vasculitis. Edema and skin rashes in both lower limbs appeared 3 days after this treatment. She was further treated for edema with furosemide and spironolactone at the same local hospital, and she was transferred to our hospital in September 2019. She did not have diabetes, nor did she have any relevant family, life, or allergy history. At admission, her vital signs on physical examination were as follows: body temperature, 36.5 °C; blood pressure, 127/90 mmHg; pulse, 71 beats/min; and respiratory rate, 20 breaths/min. Several round areas of the skin rash of varying sizes were seen on the arms, legs, and trunk. Pitting edema was observed in both lower limbs. Abdominal and cardiac exams showed no abnormalities.

**Fig. 1:**
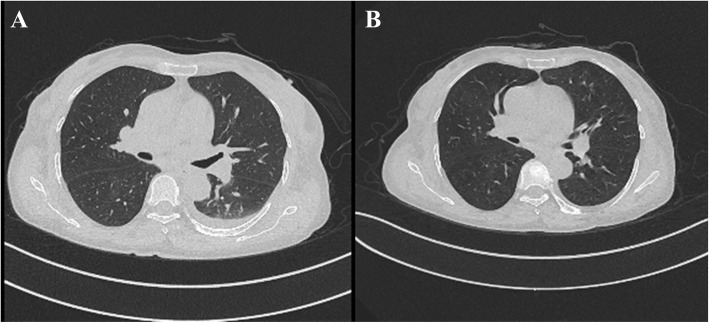
Computerized tomography (CT) images of the lungs. **a**, October 29; **b**, November 14

The laboratory test results were as follows: urinary protein, 2+; blood protein, 3+; urinary protein excretion, 5.02 g/24 h; and creatinine, 385 μmol/L (Table 1). Further assessment revealed that the patient was positive for ANCAs by indirect immunofluorescence (a titer of 1:10), positive for p-ANCAs (a titer of 3.1 as the antibody index) by enzyme-linked immunosorbent assay (ELISA), and negative for both anti-GBM and anti-proteinase 3 (c-ANCA) antibodies after three repeated assays (Table 2). Computerized tomography (CT) revealed scattered areas of inflammation in both lungs, with a small amount of pericardial effusion and bilateral pleural effusion. Half a month later, CT showed no inflammation in either lung, and the pleural effusion had disappeared (Fig. 1). Renal ultrasonography showed normal-sized kidneys with elevated echogenicity.

**Table 1:** Laboratory characteristics

Component	Value	Reference range	Interpretation
**Urinalysis results**			
Urinary protein	2+	Negative	High
Red blood Cells (n/HP)	69	0–3	High
White blood Cells (n/HP)	1	0–5	Normal
Urinary protein excretion (g/24 h)	5.02	<0.15	High
P/Cr ratio (mg/g)	3869	<30	High
Sugar sediment (mmol/L)	0	Negative	Normal
**Peripheral blood**			
Red blood cells (× 10^12^/L)	3.11	3.8–5.1	Low
Hemoglobin (g/L)	91	115–150	Low
White blood cells (×10^9^/L)	5.65	3.5–9.5	Normal
Platelets (×10^9^/L)	128	100–300	Normal
Neutrophils (×10^9^/L)	4.02	1.8–6.3	Normal
**Chemistries**			
Serum creatinine (μmol/L)	385	41–73	High
Blood urea nitrogen (mmol/L)	22.3	2.6–7.5	High
Uric acid (μmol/L)	521	160–380	Normal
eGFR (ml/min/1.73 m^2^)	11.27	56–122	Low
Total protein (g/L)	54.2	65–85	Low
Serum albumin (g/L)	38.4	40–55	Low
Na (mmol/L)	138.5	137–147	normal
K (mmol/L)	5.63	3.5–5.3	High
Ca (mmol/L)	1.89	2.11–2.52	Low
P (mmol/L)	0.90	0.85–1.51	normal
C-reactive protein (mg/L)	3.91	<5	Normal
PCT (ng/ml)	0.14	<0.046	High

Abbreviations: P/C ratio, ALB/Cr (urinary), *eGFR* estimate glomerular filtration rate, *PCT* procalcitonin

**Table 2:** Serological data for antibodies

	ANCA	p-ANCA	c-ANCA	anti-GBM
Reference range	Negative	<1.0	<1.0	<2.0
2019/10/15	pANCA(+)(1:10)	3.1	<1.0	<2.0
2019/10/29	pANCA(+)(1:10)	<1.0	<1.0	<2.0
2019/11/15	Negative	<1.0	<1.0	<2.0

Abbreviations: *ANCA* Anti-neutrophil cytoplasmic antibodies, *p-ANCA* Anti-myeloperoxidase ANCA, *c-ANCA* Anti-proteinase 3 ANCA, *anti-GBM* anti-glomerular basement membrane antibodies

**Fig. 2:**
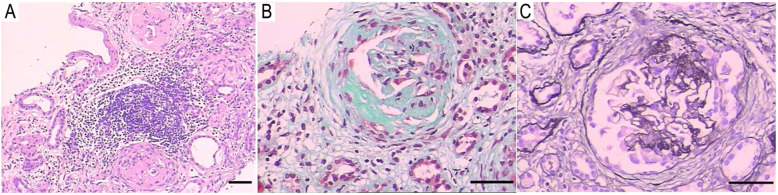
Light microscopy. **a**, Hematoxylin and eosin (HE) staining showed globally sclerotic glomeruli and the infiltration of inflammatory cells (× 100); **b**, Masson’s trichrome staining showed a large circumferential crescent (Masson × 200); **c**, The use of periodic acid-silver methenamine (PASM) stain identified the rupture of the glomerular basement membrane (GBM) and a small circumscribed crescent (× 200). Scale bars = 50 μm

**Fig. 3:**
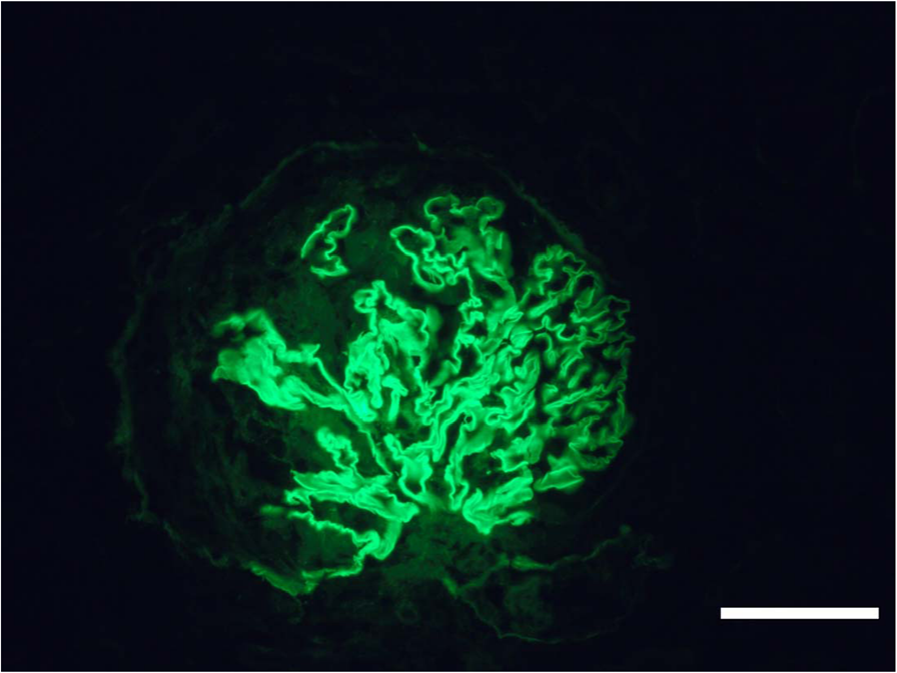
Immunofluorescence. Immunofluorescence for immunoglobulin G revealed linear deposits along the GBM. Scale bars = 50 μm

**Fig. 4:**
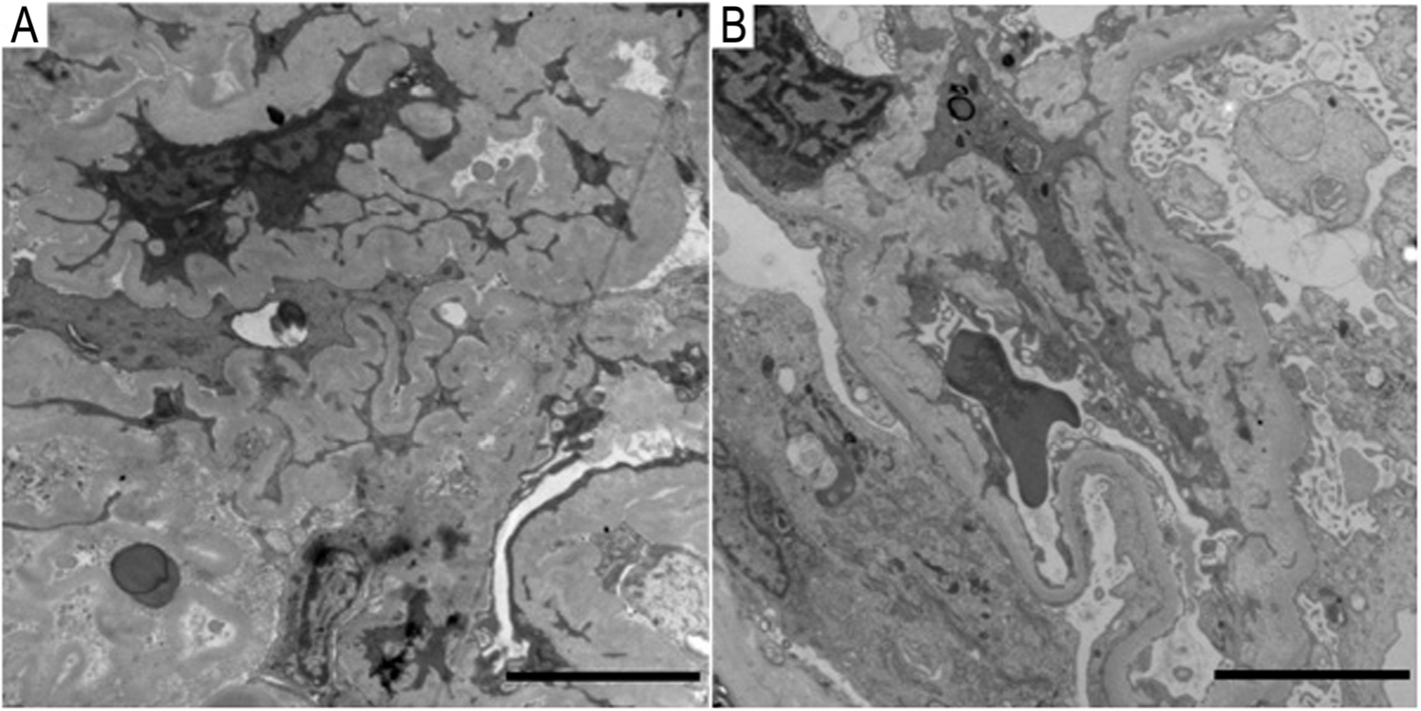
Electron microscopy. Electron microscopy findings showed the collapse and rupture of the GBM with the presence of foot process fusion. The absence of electron-dense deposits was another EM hallmark of the disease. Scale bars = 50 μm

Light microscopy on a Nikon ECLIPSE E-200 microscope (Nikon, Tokyo, Japan) revealed a total of 13 glomeruli, with three globally sclerotic glomeruli. Ten glomeruli had cellular crescents, and three had fibrocellular crescents, which suggested crescentic glomerulonephritis. The obliteration of Bowman’s capsules and segmental sclerotic lesions were noted in 5 glomeruli. In addition, tubular atrophy (30%) was accompanied by interstitial fibrosis (30%), with the interstitial infiltration of lymphocytes, monocytes, and plasmocytes (Fig. 2a, b, c). Immunofluorescence was observed under Olympus BX41microscope (Olympus, Tokyo, Japan) to assess five glomeruli, which showed IgG linearly deposited alongside the GBM (Fig. 3). Electron microscopy (EM) performed with a transmission electron microscope (JEOL Ltd., Tokyo, Japan) showed the collapse and rupture of the GBM in the presence of foot process fusion. The absence of electron-dense deposits was another EM hallmark of the disease (Fig. 4a, b). Therefore, a diagnosis of anti-GBM disease with positivity for p-ANCA and another atypical ANCA was established, despite repeated negative serum anti-GBM antibody test results.

After the diagnosis of anti-GBM disease was established based on the clinical features and biopsy findings in our patient, treatment with methylprednisolone and plasmapheresis was initiated immediately. The treatment was initiated with 500 mg of intravenous high-dose methylprednisolone, which was administered three times, followed by oral prednisone at 50 mg per day. A hemodialysis catheter was implanted in the right femoral vein to enable plasmapheresis every 2 days (ten sessions in all). Interestingly, positivity for p-ANCA disappeared, but the patient was still positive for total ANCAs after the fourth session of plasmapheresis; the patient was negative for both c-ANCA and anti-GBM antibodies at admission. However, no signals for total ANCA or anti-GBM antibodies were observed after ten sessions of plasmapheresis.

The patient had good tolerance of all drugs and procedures and was successfully discharged after medical care. She was followed up via outpatient visits to continue treatment with prednisolone and cyclophosphamide. Additionally, her kidney function and lung disease were regularly monitored in specialized departments.

## Discussion and conclusion

Anti-GBM disease represents a rarely encountered pulmonary-renal disorder that manifests as the rapid progression of glomerulonephritis and alveolar hemorrhage. The pathogenesis of anti-GBM disease involves anti-GBM antibodies in the blood that target the α-3 chain of collagen IV in the glomerular and/or alveolar basement membranes [3]. An elevated serum level of anti-GBM antibodies guides the diagnostic process. However, a tissue biopsy sample showing a linear pattern of antibody deposition along the basement membranes of kidney glomeruli, with or without alveoli showing this pattern of antibody deposition, is considered an important indicator confirming the diagnosis of anti-GBM disease [4]. This is especially critical in patients with suspected anti-GBM disease whose serum is negative for anti-GBM antibodies. The patient, whose repeated anti-GBM antibody tests were negative, was positive for anti-GBM antibodies according to immunofluorescence in a renal biopsy sample. Moreover, her serum was positive for anti-MPO antibodies and another atypical ANCA. Thus, we are the first to report this unusual presentation of anti-GBM disease.

Several factors may explain the negative ELISA results in patients with anti-GBM disease. First, high-affinity autoantibodies can be trapped in the kidneys or lung, which results in amounts of anti-GBM antibodies in the blood below the level necessary for detection by ELISA, as previously reported in patients with anti-GBM antibody disease [5–7]. In addition, ELISA techniques used to detect anti-GBM antibodies could give false-negative results, despite their high sensitivity (> 95%) and specificity (> 97%) [8]. Another possibility is that the average dissociation rate constant (K_diss_) of the antibodies might be unusually high; although these antibodies can bind to the GBM, in the ELISA, they can dissociate during the washing period [7]. In our opinion, the first mechanisms described above are more likely the explanations for the negative results. Thus, a negative serum anti-GBM antibody test is not sufficient to rule out anti-GBM disease. Kidney or lung biopsy is required to confirm or exclude the diagnosis of anti-GBM disease in patients with a high level of clinical suspicion whose serum is negative for anti-GBM antibodies.

Crescentic glomerulonephritis in the setting of elevated ANCA and anti-GBM titers is referred to as a double-positive disease. Approximately one-third of all patients with anti-GBM antibodies are also positive for ANCAs, generally myeloperoxidase ANCAs [9]. A multicenter study that included 646 patients from four centers in three European countries showed that 5.7% of patients were double-positive, with 70% of them positive for p-ANCA [10]. There are currently no reports of positivity for atypical ANCAs in patients with anti-GBM disease. The present patient was diagnosed with anti-GBM disease on the basis of the presence of anti-GBM antibodies in a kidney biopsy sample and anti-MPO antibodies and atypical ANCAs in a blood sample. Patients with double-positive disease have extrarenal signs comparable to those of patients with ANCA-associated vasculitis. At the same time, the renal symptoms of double-positive disease mimic those of anti-GBM disease at the clinical and histological levels [11]. Similarly, the renal signs in our patient were hematuria, proteinuria, elevated serum creatinine, and pathological changes in biopsy samples indicative of anti-GBM disease. In contrast, the extrarenal manifestations were ankle pain and areas of skin rash on the arms, limbs and trunk, which are indicative of ANCA-associated vasculitis.

Therapy for double-positive anti-GBM disease is comparable to that for single-positive anti-GBM disease, which involves high-dose glucocorticoids, cyclophosphamide, and plasmapheresis [4]. Once the diagnosis of anti-GBM disease is established, plasmapheresis should be performed to remove circulating pathogenic antibodies. At the same time, cyclophosphamide and prednisone are recommended to prevent further autoantibody production and attenuate the inflammation. Plasmapheresis and methylprednisolone were initiated immediately in our patient after the diagnosis of anti-GBM disease was made on the basis of biopsy. The circulating autoantibodies were cleared after ten sessions of plasmapheresis.

A few limitations of our study are important to mention. First, we did not carry out an immunoassay to quantitate MPO because there was no extra tissue available. Second, an insufficient amount of the patient’s serum was available, which meant that a Western blot for anti-GBM antibodies could be performed. Finally, the reason two antibodies, p-ANCA and atypical ANCA, appeared in the same serum sample may need further exploration.

In conclusion, cases of anti-GBM disease in patients whose serum is negative for anti-GBM antibodies and positive for ANCAs are clinically sporadic. The use of ELISA to detect anti-GBM antibodies can yield false-negative results. Thus, negative serum anti-GBM antibody test results are not sufficient to rule out anti-GBM disease. Kidney or lung biopsy is needed to confirm or exclude anti-GBM disease in patients with a high degree of clinical suspicion whose serum is negative for anti-GBM antibodies. Insights from this unusual case might help physicians diagnose a rare form of glomerulonephritis and treat affected patients in a timely manner.

## Data Availability

The datasets used in this study are available from the corresponding author on reasonable request.

## Abbreviations

Anti-GBM: Anti-Glomerular Basement Membrane
ANCA: Anti-Neutrophil Cytoplasmic Antibody
MPO: Myeloperoxidase
p-ANCA: Anti-Myeloperoxidase ANCA
c-ANCA: Anti-Proteinase 3 ANCA
ELISA: Enzyme-Linked Immunosorbent Assay
CT: Computerized Tomography
IgG: Immunoglobulin G
